# *Drosophila* screen connects nuclear transport genes to DPR pathology in c9ALS/FTD

**DOI:** 10.1038/srep20877

**Published:** 2016-02-12

**Authors:** Steven Boeynaems, Elke Bogaert, Emiel Michiels, Ilse Gijselinck, Anne Sieben, Ana Jovičić, Greet De Baets, Wendy Scheveneels, Jolien Steyaert, Ivy Cuijt, Kevin J. Verstrepen, Patrick Callaerts, Frederic Rousseau, Joost Schymkowitz, Marc Cruts, Christine Van Broeckhoven, Philip Van Damme, Aaron D. Gitler, Wim Robberecht, Ludo Van Den Bosch

**Affiliations:** 1 KU Leuven - University of Leuven, Department of Neurosciences, Experimental Neurology and Leuven Research Institute for Neuroscience and Disease (LIND), B-3000 Leuven, Belgium; 2 VIB, Vesalius Research Center, Laboratory of Neurobiology, B-3000 Leuven, Belgium; 3 Department of Molecular Genetics, VIB, B-2610 Antwerp, Belgium; 4 Institute Born-Bunge, University of Antwerp, B-2610 Antwerp, Belgium; 5 Department of Neurology, University Hospital Ghent and University of Ghent, B-9000 Ghent, Belgium; 6 Department of Genetics, Stanford University School of Medicine, Stanford, CA 94305 USA; 7 Switch Laboratory, VIB, B-3000 Leuven, Belgium; 8 Switch Laboratory, Department of Cellular and Molecular Medicine, KU Leuven, B-3000 Leuven, Belgium; 9 VIB Laboratory of Systems Biology, Gaston Geenslaan 1, B-3001 Leuven, Belgium; 10 KU Leuven - University of Leuven, Department of Microbial and Molecular Systems, Laboratory for Genetics and Genomics, Gaston Geenslaan 1, B-3001 Leuven, Belgium; 11 KU Leuven - University of Leuven, Department of Human Genetics, Laboratory of Behavioral and Developmental Genetics, B-3000 Leuven, Belgium; 12 VIB Center for the Biology of Disease, B-3000 Leuven, Belgium; 13 University Hospitals Leuven, Department of Neurology, B-3000 Leuven, Belgium

## Abstract

Hexanucleotide repeat expansions in *C9orf72* are the most common cause of amyotrophic lateral sclerosis (ALS) and frontotemporal degeneration (FTD) (c9ALS/FTD). Unconventional translation of these repeats produces dipeptide repeat proteins (DPRs) that may cause neurodegeneration. We performed a modifier screen in *Drosophila* and discovered a critical role for importins and exportins, Ran-GTP cycle regulators, nuclear pore components, and arginine methylases in mediating DPR toxicity. These findings provide evidence for an important role for nucleocytoplasmic transport in the pathogenic mechanism of c9ALS/FTD.

*C9orf72* hexanucleotide repeat expansions are the most common genetic cause of ALS and FTD (c9ALS/FTD)^1^,^2^. Despite the importance of these mutations, the underlying pathogenic mechanisms remain elusive. Three main hypotheses have been proposed to explain how *C9orf72* mutations could cause disease: haploinsufficiency due to lowered transcription of the *C9orf72* gene, RNA toxicity resulting from the sequestration of essential RNA-binding proteins by sense and antisense repeat RNA foci that accumulate in the nucleus and cytoplasm, and repeat associated non-ATG (RAN) translation of sense and antisense RNA^3^. This unconventional form of translation results in the generation of distinct aggregation-prone dipeptide repeat proteins (DPRs). These DPRs are found in the hallmark p62-positive, TDP-43 negative inclusions seen in c9FTD/ALS patients^4^,^5^,^6^. Mounting evidence points to a direct role of these DPRs in neurodegeneration^7^,^8^,^9^,^10^ but the mechanism by which these DPRs cause toxicity remains unresolved and of intense interest. Defining the cellular mechanisms of DPR toxicity is necessary to understand the disease pathogenesis and may reveal new targets for therapeutic intervention.

We used *Drosophila* to investigate the mechanisms by which *C9orf72* DPRs cause toxicity and neurodegeneration. To focus specifically on DPR toxicity, we had to experimentally separate the generation of the DPRs from the presence of repeat RNA. We generated expression constructs allowing ATG-mediated expression of a single DPR and codon-optimized these constructs to reduce the formation of stable repeat RNA secondary structures. Hence, these constructs allow us to attribute observed phenotypes solely to expression of one DPR, and rule out any confounding RNA toxicity or RAN translation. We generated fly lines expressing 50 repeats of four of the five possible DPRs (GA, GR, PA and PR). Consistent with recent reports^8^,^9^,^10^, we found that expression of the arginine-rich DPRs, GR and PR, strongly reduced survival in flies expressing these DPRs in adult flies either ubiquitously or motor neuron-specific (Fig. S1). Thus, this *Drosophila* model recapitulates robust *C9orf72* DPR toxicity, providing a tractable system to identify and characterize toxicity modifier genes.

Jovičić *et al*. reported results from two unbiased genome wide screens in yeast for suppressors and enhancers of PR toxicity^11^. These two screens identified a striking number of modifier genes involved in nucleocytoplasmic transport. These modifiers include karyopherins, nuclear pore complex components, and enzymes involved in generating the Ran-GTP gradient that drives nuclear transport. Since the general principles and key molecules of nucleocytoplasmic transport are highly-conserved from yeast to flies to humans^12^, we sought to validate these results in an animal model and to test the hypothesis that genes involved in nuclear transport could also modify *C9orf72* DPR toxicity *in vivo*. We therefore performed a targeted RNAi screen in *Drosophila*. To facilitate the rapid identification of modifiers of DPR toxicity, we used eye degeneration as a readout. We directed expression of a single copy of the 25 PR repeat construct to the fly eye. This caused a moderate degenerative phenotype (Fig. 1a), providing the ability to identify both suppressors and enhancers.

To focus on nucleocytoplasmic transport and related processes, we compiled a library of 121 independent RNAi lines targeting 55 fly genes (Table S1), encoding nuclear pore complex proteins, importins, exportins, regulators of the Ran-GTP cycle, and arginine methylases, which affect protein localization by modulating NLS sequences^13^. We expressed each RNAi together with the PR25 construct and scored for the ability to enhance or suppress the phenotype (Fig. 1b, Fig. S2). We identified 15 enhancers and 4 suppressors of the PR25 eye phenotype (Table 1, Table S4). Importantly, the RNAi lines did not cause a degenerative eye phenotype in a wild type background (Fig. S3a) and the RNAi lines that suppressed PR toxicity did not affect PR expression (Fig. S3b).

Knockdown of four members of the importin family (Ranbp11, Kap-alpha3, Fs(2)Ket and Trn), which mediate nuclear import of cargo proteins, caused striking enhancement of the PR25 eye phenotype (Fig. 1c). Knockdown of another importin, CG32165, mildly suppressed toxicity and knockdown of the exportin emb enhanced PR toxicity. We also identified the two regulators of the Ran-GTP cycle (Rcc1 and RanGap) as modifiers of PR25 toxicity (Fig. 1d). Subunits of the nuclear pore complex were suppressors (Nup50, Nup107 and Nup154) and enhancers (Mtor, Nup44A, Nup62 and Nup93-1) (Fig. 1e). Finally, knockdown of four different arginine methyltransferases (Art1, Art6, Art7 and Fbx011) enhanced PR toxicity.

The strongest enhancer of PR-mediated neurodegeneration was knockdown of Trn (Fig. 2a, Fig. S4), the fly ortholog of TNPO1, encoding transportin 1. Of notice, the yeast TNPO1 homolog Kap104 was one of the strongest suppressors of PR toxicity in yeast^11^. Interestingly, transportin 1 has been connected to another form of ALS/FTD, namely related to FUS^13^,^14^. ALS-causing mutations in FUS/TLS impair transportin-mediated nuclear import, resulting in FUS cytoplasmic accumulation and aggregation^15^ and in FTD cases with FUS pathology transportin 1 is mislocalized to cytoplasmic FUS aggregates^14^. To determine whether PR could directly act on transportin 1 function, we performed computational docking simulations (Fig. 2b) and predicted that PR can interact with transportin 1. This suggested that PR might compete for endogenous transportin 1 cargoes. To test this hypothesis, we analyzed the effect of PR expression on the localization of a well-characterized transportin 1 cargo, the neuronal RNA-binding protein Elav. We observed increased cytoplasmic localization and decreased nuclear staining of Elav in flies expressing PR (Fig. 2c). Moreover, Elav mislocalization increased further upon Trn knockdown (Fig. 2c). Consistent with previous reports, we also observed the cytoplasmic aggregation of the transportin 1 cargo hnRNPA3 in human c9FTD (Fig. 2d, Table S2). These findings suggest a potential role of transportin 1 in the pathogenesis of c9ALS/FTD.

Finally, knockdown of four out of ten arginine methyltransferase genes enhanced PR toxicity in *Drosophila* (Table 1, Fig. 2e). One of these, PRMT1, has been previously implicated in ALS caused by FUS/TLS mutations^13^,^16^. To determine whether this enzyme could directly affect DPR toxicity, we performed colocalization experiments in cell lines. PRMT1 colocalized with both GR and PR (Fig. 2f, Fig. S5). We subsequently tested several commercially available antibodies to detect methylation. One of them, ASYM24, was able to detect asymmetric arginine dimethylation of GR, but not PR. This was not surprising since this antibody was originally raised against a peptide showing strong sequence similarity with GR, but not PR (Fig. S5). Using this antibody we found GR accumulations to be methylated in transfected cells, but also an association of GR with other methylated proteins, as determined by super resolution microscopy (Fig. 2g). To validate this new aspect of DPR pathology in humans, we performed immunostaining on c9FTD brain samples and detected abundant methylated inclusions (Fig. 2h, Table S3). These results implicate arginine methyltransferases to c9ALS/FTD pathogenesis.

In summary, we performed a targeted genetic modifier screen in *Drosophila* and confirm results obtained in two yeast screens^11^. Both studies consolidate DPRs as major contributing factors to c9 toxicity and point at a role for nucleocytoplasmic transport in this toxicity. Strikingly, the homologs of the strongest genes from the yeast screens were potent modifiers of DPR toxicity in *Drosophila*. Not only does this validate the effectiveness of modifier genes discovered in yeast in an animal model, it also suggests that DPR pathologies associated with c9ALS/FTD disrupt a highly conserved facet of cell biology.

Depletion of RNA-binding proteins (RBPs) such as TDP-43, FUS and others from the nucleus, and cytoplasmic accumulation are pathognomonic features of both ALS and FTD^17^, including c9ALS/FTD^18^,^19^,^20^. The upstream triggers of this nuclear depletion and subsequent cytoplasmic aggregation are unresolved. Our results help to explain how disturbances in nucleocytoplasmic trafficking caused by *C9orf72* repeat expansion pathology could trigger RBP mislocalization and subsequent aggregation. Most RBPs are tightly regulated in their cellular localization, and shuttle in a strictly controlled manner between nucleus and cytoplasm. Impairments, even subtle, to the nuclear pore, karyopherins, or the Ran-GTP gradient, could perturb this sensitive equilibrium providing a first hit that would eventually lead to aberrant accumulation of RBPs in the cytoplasm, setting off a cascade of aggregation and sequestration of these proteins into pathological inclusions. Interestingly, preliminary histopathological studies suggest that *C9orf72* DPR accumulation predates TDP-43 pathology in FTLD patients harboring *C9orf72* mutations^21^,^22^.

DPR pathology is one way that *C9orf72* mutations might contribute to disease pathogenesis. First of all, a role for *C9orf72* loss of function, owing to decreased expression of *C9orf72*, has not been conclusively ruled in or out. A recent report potentially even links the C9ORF72 protein to nuclear transport^23^. Secondly, sense and antisense RNA transcripts produced from the GGGGCC hexanucleotide repeat expansion accumulate in the nucleus and cytoplasm of mutation carriers^3^ and could cause disease by an RNA toxicity mechanism (e.g., by sequestering important regulatory proteins like splicing factors and other RNA-binding proteins). Two recent studies report the results from genetic screens similar to ours using GGGGCC repeat fly models^24^,^25^. Compellingly, these studies also identify nuclear transport as a key pathogenic factor in these fly models, hereby suggesting that the repeat RNA itself could also directly perturb nuclear transport. However, two other reports have ruled out RNA toxicity in flies, at least at the short lengths used in the current models, and attribute all observed phenotypes to DPR toxicity^10^,^26^. Moreover, since we identify similar modifiers using a pure DPR model, this raises the question whether the repeat RNA itself is truly involved in the observed nuclear transport defects.

The three potential pathogenic mechanisms are not mutually exclusive and future studies will be required to disentangle the relative contributions of DPR proteotoxicity, RNA toxicity, and *C9orf72* loss of function to c9FTD/ALS. Moreover, why the same *C9orf72* mutations cause dementia in some patients, ALS in others patients, and ALS/FTD in yet others, even within the same family, is unresolved and might be influenced by modifier genes. A better understanding of the mechanisms by which *C9orf72* hexanucleotide repeats cause disease will allow the identification of novel targets for therapeutic intervention.

## Material & Methods

### Plasmids and strains

FLAG-tagged DPR expression constructs were designed by manually codon-optimizing the sequence, and using Mfold software^27^ to control for any persistent stable secondary structures (Fig. S6, Table S5). DNA constructs were synthesized by Genscript (Piscataway, USA). DPR constructs were subcloned in CMV6 entry plasmids (Origene) for expression in mammalian cells. Subcloning to the pUAST-attB backbone allowed the generation of transgenic fly lines by targeted insertion into the 62E1 attP locus on the third chromosome (GenetiVision, USA). The PRMT1-EGFP construct was a kind gift of Dr. F. Fackelmayer (Laboratory for Epigenetics and Chromosome Biology, Ioannina, Greece).

### Lifespan assay

Lifespan experiments were performed using, the TARGET system^28^. The tub-Gal4 or the D42-Gal4 driver was combined with a ubiquitously expressed temperature-sensitive Gal80 inhibitor (tub-Gal80ts). Fly crosses were grown at 18 °C and adult progeny of carrying the tub-Gal4 or the D42-Gal4 and tub-Gal80 chromosomes and the UAS-DPR responder gene were shifted to 29 °C to allow expression of the transgenes. Females were collected within 24 hr of eclosion and grouped into batches of 10 flies per food vial. Fresh food vials were provided every 2–3 days.

### Dot blot analysis

The GMR PR25 screening stock was crossed with the four suppressor lines and offspring was collected. 30 flies per condition were decapitated and fly heads were homogenized in 30 μl of RIPA buffer. 15 μl of homogenate was spotted onto a nitrocellulose membrane (Amersham) and air dried. Loading controls were visualized using Coomassie staining (Life Technologies). Membranes were blocked in 5% milk powder (Bio-Rad) in TBS-T buffer and probed with a custom rabbit PR antibody (Thermo Scientific). HRP-labeled anti rabit secondary antibody was used (Dako) and membranes were imaged using chemiluminescence (Pierce) and an Image Quant Las 4000 imaging station (GE).

### Cell culture and transfection

HeLa cells (ATCC) were cultured in high glucose DMEM (Invitrogen) supplemented with 10% fetal bovine serum (Greiner), 4 mM Glutamax (Invitrogen), penicillin (100 U/mL), streptomycin (100 μg/mL) and non-essencial amino acids (1%). Neuroblastoma cells (SH-SY5Y cells, ATCC) were cultured in high glucose DMEM/F12 (Invitrogen) supplemented with 10% fetal bovine serum (Greiner), 4 mM Glutamax (Invitrogen), penicillin (100 U/mL), streptomycin (100 μg/mL) and non-essencial amino acids (1%). Cells were grown at 37 °C in a humidified atmosphere with 7% CO2. Cells were transiently transfected using Lipofectamine 3000 (Invitrogen) according to manufacturer’s instructions.

### Immunofluorescence, immunohistochemistry and microscopy

Fly brains were dissected and fixed in 4% formaldehyde in PBS. Cells were fixed 24 h after transfection in 4% formaldehyde in PBS and stained according standard protocols. Following antibodies were used: anti-FLAG (F3165, Sigma), rabbit anti-FLAG (#2368S, Cell Signaling), rabbit anti-PR (custom generated antibody, Thermo Scientific), ASYM24 (07-414, Millipore), rat anti-Elav (7E8A10, Developmental Studies Hybridoma Bank). AlexaFluor 555 and AlexaFluor 488 secondary antibodies (Life Technologies) were used. Nuclei were visualized using Hoechst counterstaining (Sigma). Slides were mounted using ProLong Gold antifade reagent (Life Technologies).

The ASYM24 antibody was originally generated against an interrupted GR repeat (KGRGRGRGRGPPPPPRGRGRGRG). This antigen shows strong sequence similarity with GR and hence was able to detect GR methylation, but not PR methylation. The antibody was specific for methylated residues as staining was abolished using a general methylation inhibitor Adox.

Confocal images were obtained using a Zeiss LSM 510 Meta NLO confocal microscope and SIM microscopy was performed using a Zeiss Elyra S.1 microscope (Carl Zeiss, Germany). SIM calculations were performed using default settings. Images were analyzed, formatted and quantified with FIJI and ImageJ software. For all experiments representative photographs are shown from multiple wells from transfections with at least two cell passages, or from multiple fly brains.

Autopsied brains of six C9orf72 carriers and two non-disease controls were obtained using informed consents and protocols that were approved by the Ethical Committee of University of Antwerp and Antwerp University Hospital and stored in the Antwerp Biobank of the Institute Born-Bunge. Methods were carried out in accordance with the approved guidelines. Clinical data are shown in Table S6. After a fixation period of 8 to 16 weeks in 10% buffered formalin, 5 μm slices were cut from following regions: frontal cortex, hippocampus with dentate gyrus and parahippocampal gyrus, cerebellar cortex, medulla oblongata. Sections were deparaffinized, rehydrated and pretreated with citric acid 0.1 M. Immunohistochemical analysis was performed with anti-hnRNPA3 antibody (AV41195, Sigma) and ASYM24 antibody (07-414, Millipore). Sections were counterstained with hematoxylin and images were taken on an Axioskop 50 light microscope (Zeiss) equipped with a CCD UC30 camera (Olympus Inc.).

### RNAi modifier screen

To identify modifiers of our PR25 eye phenotype we crossed 121 RNAi lines with our screening stock. The RNAi lines were obtained from VDRC or Bloomington Drosophila stock center (USA). For each cross the collected offspring was divided by sex, and the genotypes were counted according to the balancers. The offspring ratio was determined by (expected offspring/counted offspring). For each sex we subsequently assigned an average color using following scoring scale (white = 1, yellow = 2, yellow-orange = 3, orange-yellow = 4, orange = 5, orange-red = 6, red-orange = 7, red = 8). Afterwards, each fly was individually scored for the presence of necrotic spots using following scoring scale (not affected = 0, mild = 1, medium = 2, heavy = 3, extreme = 4). We crossed each line at least two independent times. After compiling all data, two researchers assigned independently a status to each RNAi line (no effect, enhancer or suppressor) as compared to a cross of the screening stock to the RNAi w1118 background. Only whenever a gene was represented by at least two RNAi lines showing a similar effect on the phenotype, the gene was classified as an enhancer or suppressor. When shown, statistics were carried out using Prism software.

RNAi lines did not present with a degenerative eye phenotype by themselves. Two RNAi lines used had a ‘greasy’ eye phenotype reminiscent of mitochondrial eye phenotypes. These lines indeed had reported off target effects on genes involved in eye development: i.e. v105181 off target CG8085, v36103 off target CG4389. Importantly, both these hits were verified by independent RNAi lines without this ‘greasy’ eye phenotype.

### Structural modeling

We employed the FoldX force field^29^ to model PR and GR in the binding pocket of transportin-1 (PDB code: 2OT8). In addition, we performed an unrestrained energy minimization using the YASARA2 force field to optimize the interactions between the PR/GR peptide and the binding pocket of transportin 1^30^. The graphical representation was generated using the Yasara program (version 13.2.21) where negatively charged residues in the binding pocket, within a distance of 5 Angstrom of the peptide, were colored in blue.

## Additional Information

**How to cite this article**: Boeynaems, S. *et al*. *Drosophila* screen connects nuclear transport genes to DPR pathology in c9ALS/FTD. *Sci. Rep*. **6**, 20877; doi: 10.1038/srep20877 (2016).

## Supplementary Material

Supplementary Information

Supplementary Table S1

## Figures and Tables

**Figure 1:**
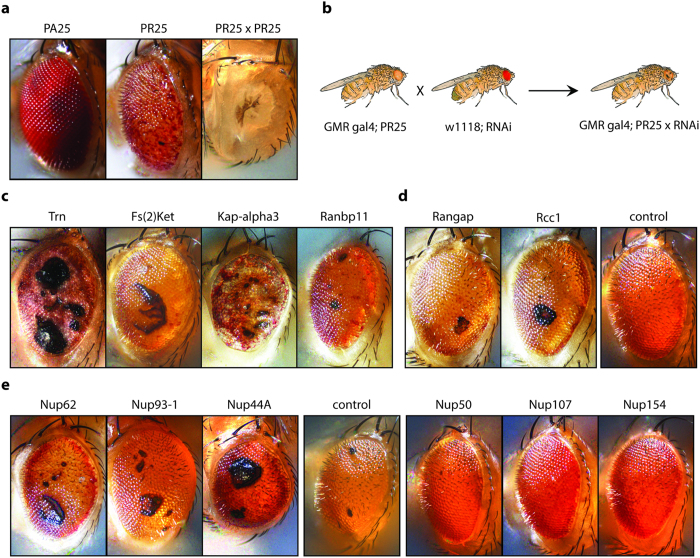
Genes implicated in nuclear transport are potent modifiers of PR toxicity in *Drosophila*. (**a**) Expression of a PR25 construct in the fly eye induces a moderate degenerative eye phenotype compared to the eyes of non-affected PA-expressing flies or severely affected flies expressing two copies of the PR25 transgene. (**b**) Setup of RNAi screen for PR25 modifiers. (**c**) Knockdown of four karyopherins (Trn, Fs(2)Ket, Kap-alpha3 and Ranbp11) strongly enhanced PR toxicity. (**d**) Knockdown of RanGTP cycle regulators Rangap and Rcc1 both enhance eye degeneration. (**e**) Knockdown of some nuclear pore complex components (Nup50, Nup107 and Nup154) suppressed degeneration others (Nup44A, Nup62, Nup93-1) enhanced the phenotype. (**a,e**) show males, (**c,d**) females.

**Figure 2:**
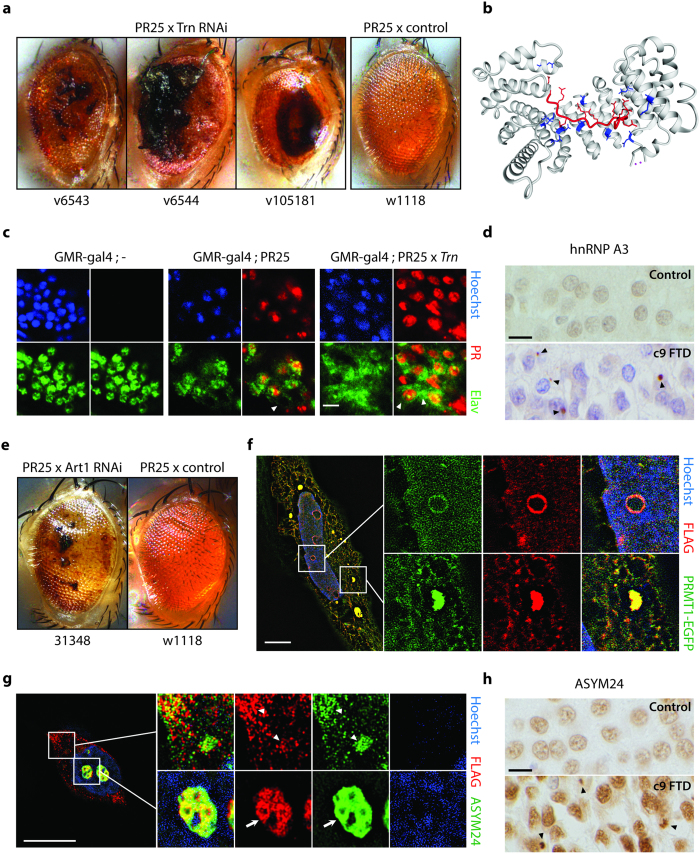
Transportin-1 and arginine methylation are directly implicated in DPR models and C9 patients. (**a**) Trn knockdown potently enhanced the PR-induced degenerative eye phenotype. Females are shown. (**b**) Computational conformational docking predictions predict that PR can fit the transportin-1 binding pocket. Positive arginine side chains of PR (red) interact with the negative side chains (blue) of the binding pocket. (**c**) Elav is mislocalized to the cytoplasm in PR expressing flies. Mislocalization is exacerbated upon Trn RNAi knockdown. Arrowheads indicate cytoplasmic staining. Scale bar indicates 5 μm. (**d**) hnRNPA3 is mislocalized in c9FTD cases (arrowheads) but not in disease-negative controls. Picture shows dentate gyrus. Scale bar indicates 10 μm. (**e**) Art1 knockdown enhances the PR-induced eye phenotype. Males are shown. (**f**) PR colocalizes with PRMT1 upon cotransfection in HeLa cells, as determined by super resolution microscopy (SIM). (**g**) GR staining associates with (arrowheads) and partially colocalizes with (arrow) ASYM24 staining in transfected neuroblastoma cells, as determined by SIM. Scale bars indicate 10 μm. (**h**) Immunostaining with ASYM24 detects methylated pathological aggregates (arrowheads) in dentate gyrus of c9FTD patient samples but not in controls. Scale bar indicates 10 μm.

**Table 1:** Modifiers of PR25-mediated eye degeneration uncovered in a targeted RNAi screen.

Fly gene	E/S	Human gene
Nuclear Pore Complex		
Mtor	E	TPR
Nup44A	E	SEH1
Nup50	S	NUP50
Nup62	E	NUP62
Nup93-1	E	NUP93
Nup107	S	NUP107
Nup154	S	NUP155
Importins/Exportins		
CG32165	S	IPO4
Fs(2)Ket	E	KPNB1
Kap-alpha3*	E	KPNA3, KPNA4
Ranbp11*	E	IPO11
Trn*	E	TNPO1
emb	E	XPO1
Ran-GTP regulators		
RanGap	E	RANGAP1
Rcc1*	E	RCC1
Arginine methytransferases		
Art1	E	PRMT1
Art6	E	—
Art7	E	PRMT7
Fbx011	E	FBX010, FBX011

^*^Indicates genes which were independently discovered in genome-wide yeast screens^11^. S = suppressor; E = enhancer.
