# Supplementary material for: Drosophila screen connects nuclear transport genes to DPR pathology in c9ALS/FTD
Source: Sci Rep. 2016 Feb 12;6:20877. doi: 10.1038/srep20877 (PMC4751451; doi:10.1038/srep20877)
Supplement: Supplementary Information [file srep20877-s1.pdf]

Supplementary Information:

Drosophila screen connects nuclear transport genes to DPR pathology in c9ALS/FTD

Steven Boeynaems, Elke Bogaert, Emiel Michiels, Ilse Gijselinck, Anne Sieben, Ana Jovičić, Greet De Baets, Wendy Scheveneels, Jolien Steyaert, Ivy Cuijt, Kevin J. Verstrepen, Patrick Callaerts, Frederic Rousseau, Joost Schymkowitz, Marc Cruts, Christine Van Broeckhoven, Philip Van Damme, Aaron D. Gitler, Wim Robberecht and Ludo Van Den Bosch

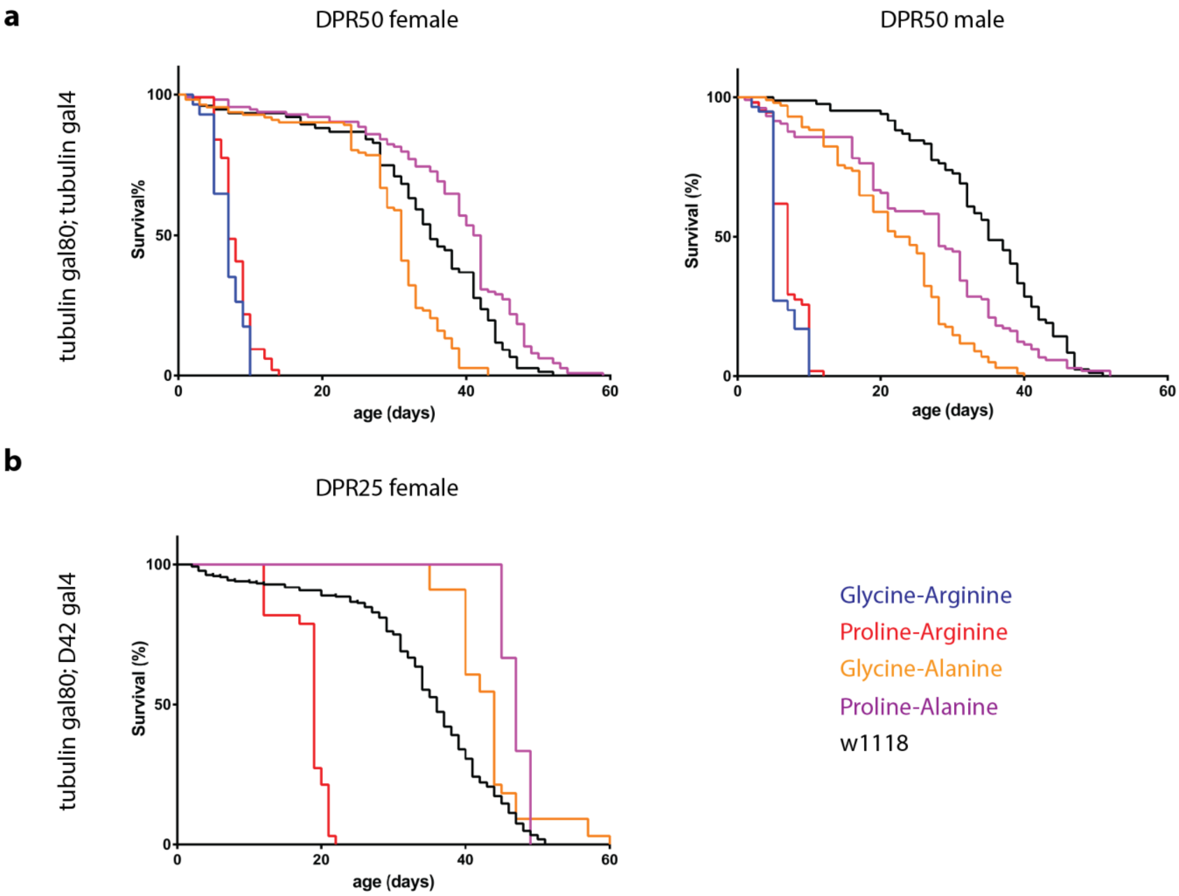

Figure S1. Arginine-rich DPRs cause degeneration and early lethality in *Drosophila*. (a) Adult-onset expression of GR50 and PR50 using a ubiquitous driver decreased survival (log-rank Mantel-Cox, $p<0.001$). (b) Adult-onset motor neuron-specific expression of PR25 also shortened lifespan (log-rank Mantel-Cox, $p<0.001$). GR25 was not available.

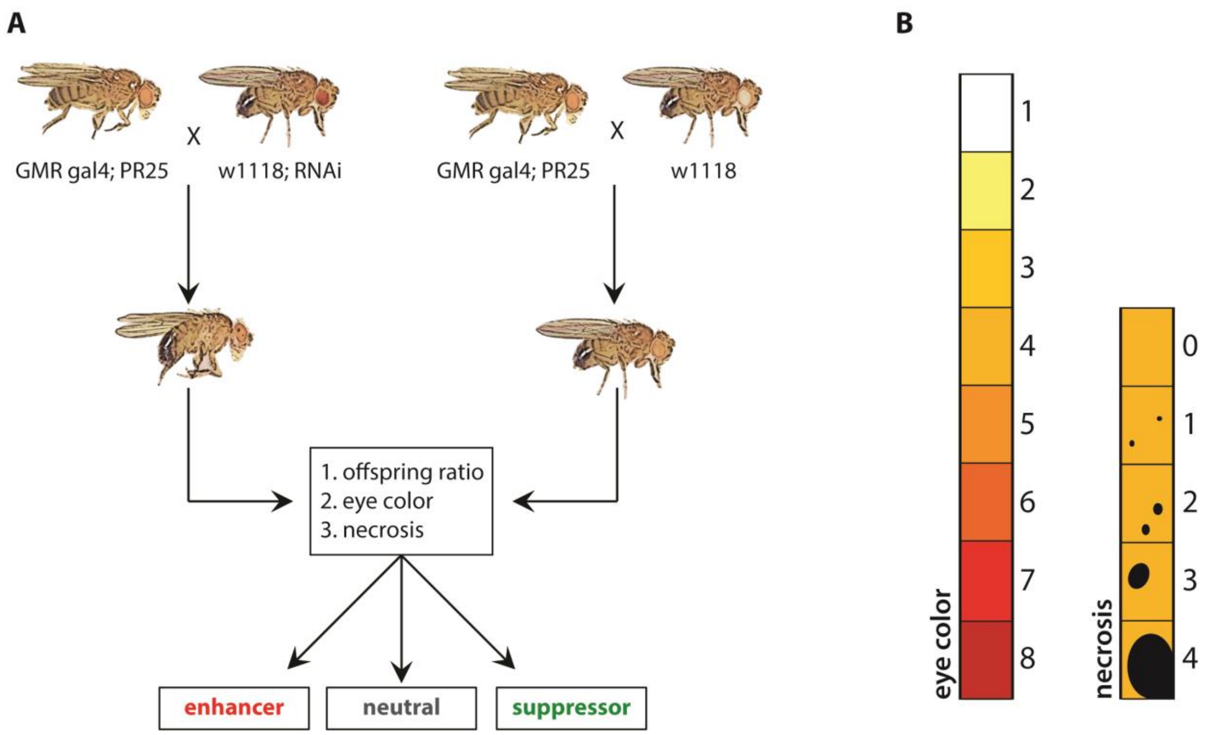

Figure S2: Set-up of PR toxicity RNAi screen. (A) General scheme indicating the overall strategy of our screen. (B) Eye color and Necrosis scoring codes for semi-quantitative evaluation of neurodegeneration.

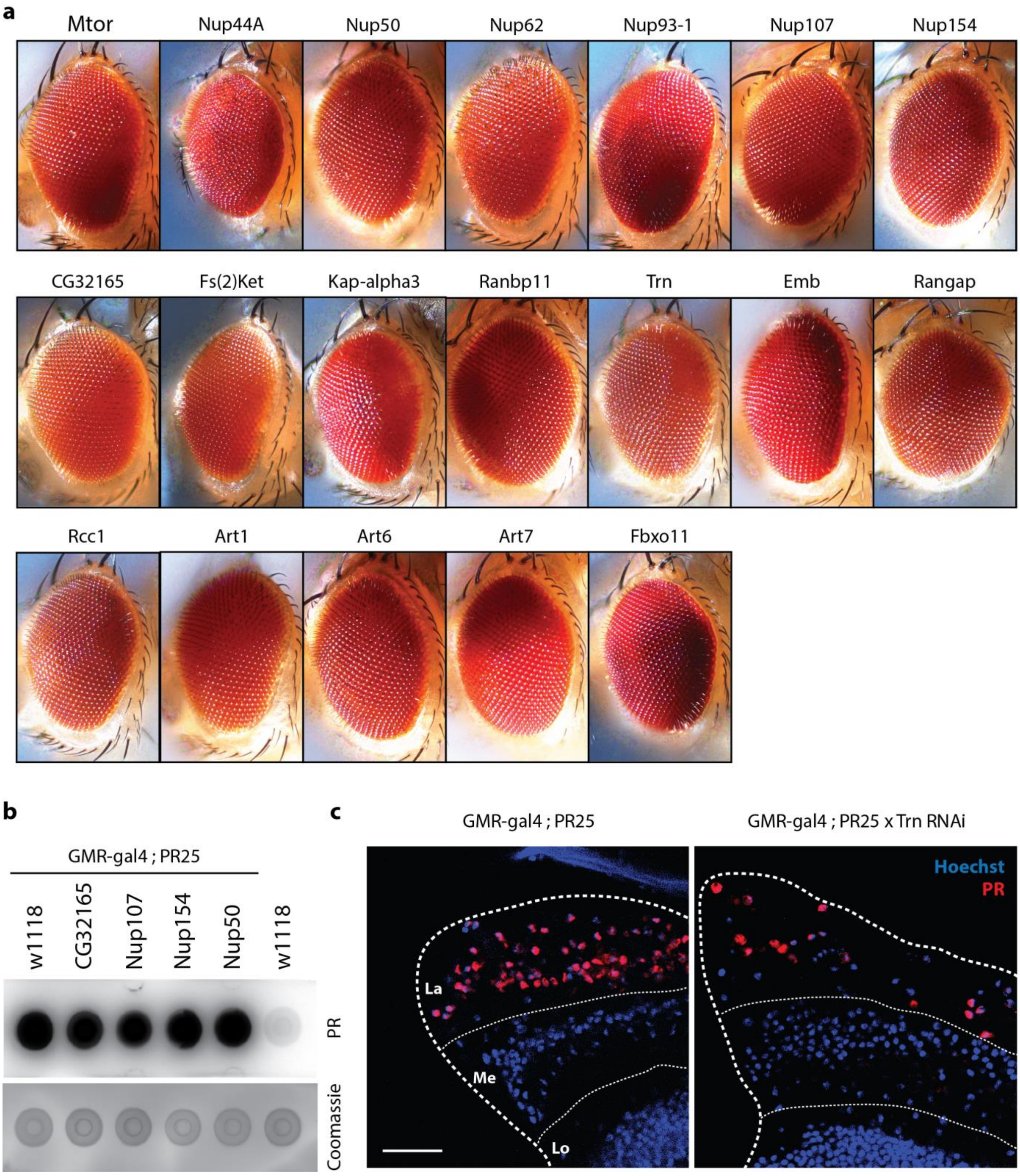

Figure S3. RNAi knockdown of the PR25 modifier genes do not cause eye degeneration in wild type flies and does not affect PR expression and localization. (a) RNAi lines that modified PR25 phenotype did not have any eye degeneration phenotype on their own. Males are shown. (b) The four identified PR25 suppressors did not affect the PR phenotype by altering its expression levels as observed by dot blot analysis on fly heads harvested from (GMR-gal4;PR25 X w1118;RNAi) offspring. (c) Crossing GMR-gal4;PR25 flies with Trn (transportin-1 ortholog) RNAi lines did not affect the predominant nuclear localization of PR in the fly retina. La – lamina, Me – medulla, Lo – lobula. Scale bar indicates 25 µm.

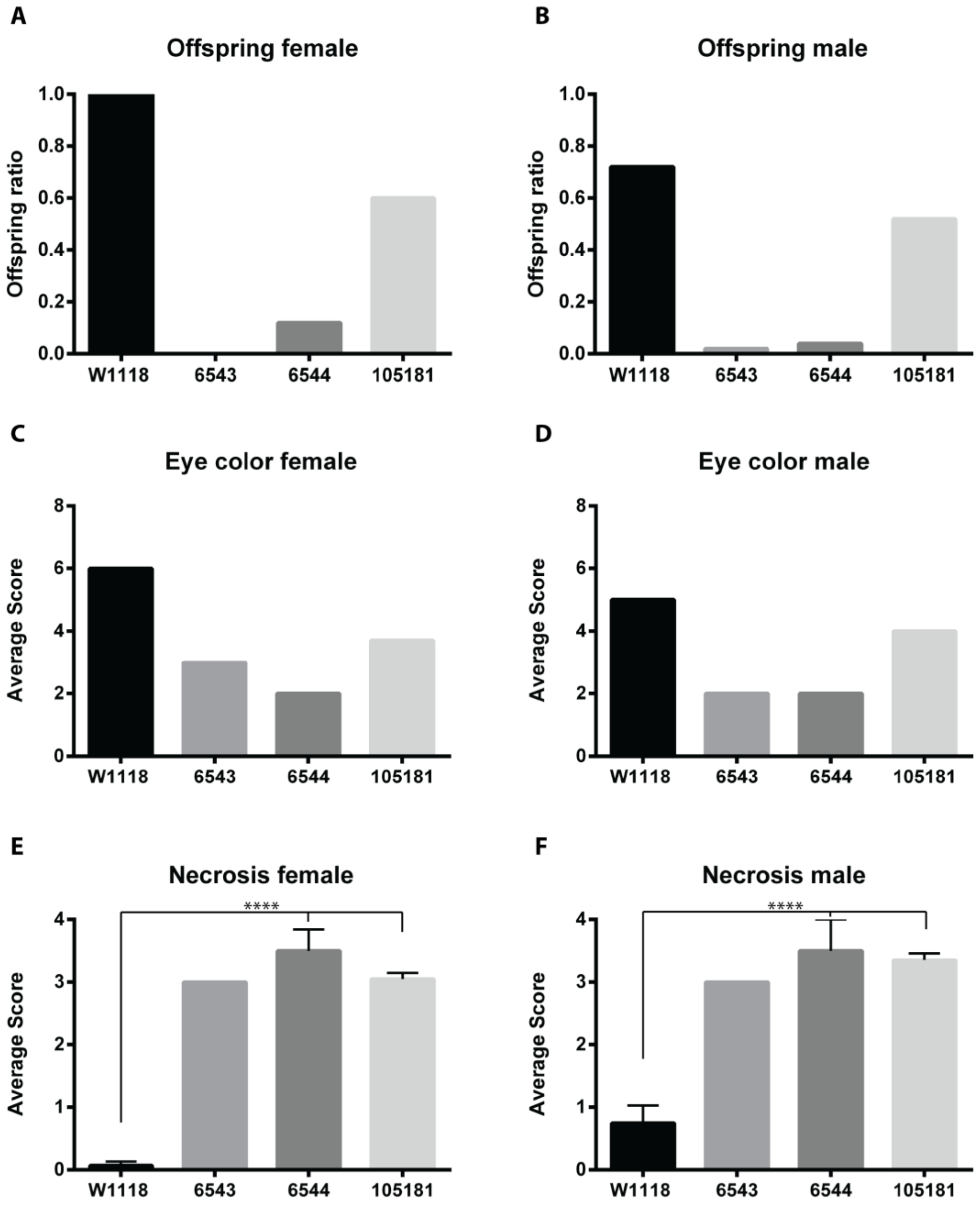

G

n	6543	6544	105181	w1118
male	132	92	119	45
female	129	110	271	45

Figure S4: Transportin-1 is a strong modifier of PR25 mediated eye degeneration. Graphs indicate offspring frequencies, average score for eye color and necrosis for three independent RNAi lines targeting Trn compared to a negative control. Results are split for males (B, D and F) and females (A, C and E). (E,F) Necrotic burden was significantly increased in RNAi flies compared to the background control (one-way Anova Kruskal-Wallis, $p<0.0001$). The number of surviving 6543 flies was too small to perform statistics (1 female, 1 male). (G) Table indicating the total number of flies counted for each set of crosses. Bar plots indicate average score (A-D) or mean +- SEM (E-F).

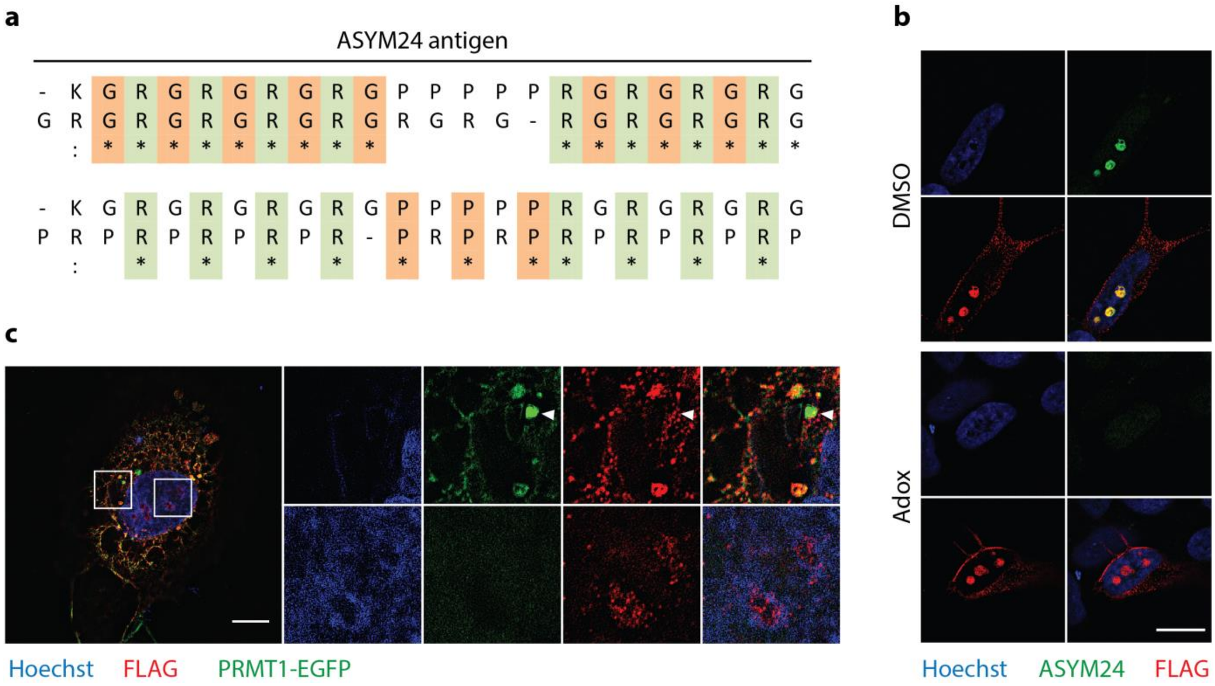

Figure S5: GR interacts with PRMT1 and its methylation is detected by ASYM24. (a) The commercially available ASYM24 antibody is predicted to recognize methylated GR but not PR, based on sequence similarity between the DPRs and its antigen. * indicates methylated residues. (b) GR methylation is detected in transfected cells. Staining is abolished in the presence of the general methylation inhibitor Adox indicating the specificity for methylated residues. Scale bar indicates 10µm. (c) GR colocalized with PRMT1 in the cytoplasm but was absent from the bright PRMT1 inclusions (arrowhead), as opposed to PR. Also no nucleolar PRMT1-PR colocalization was observed.

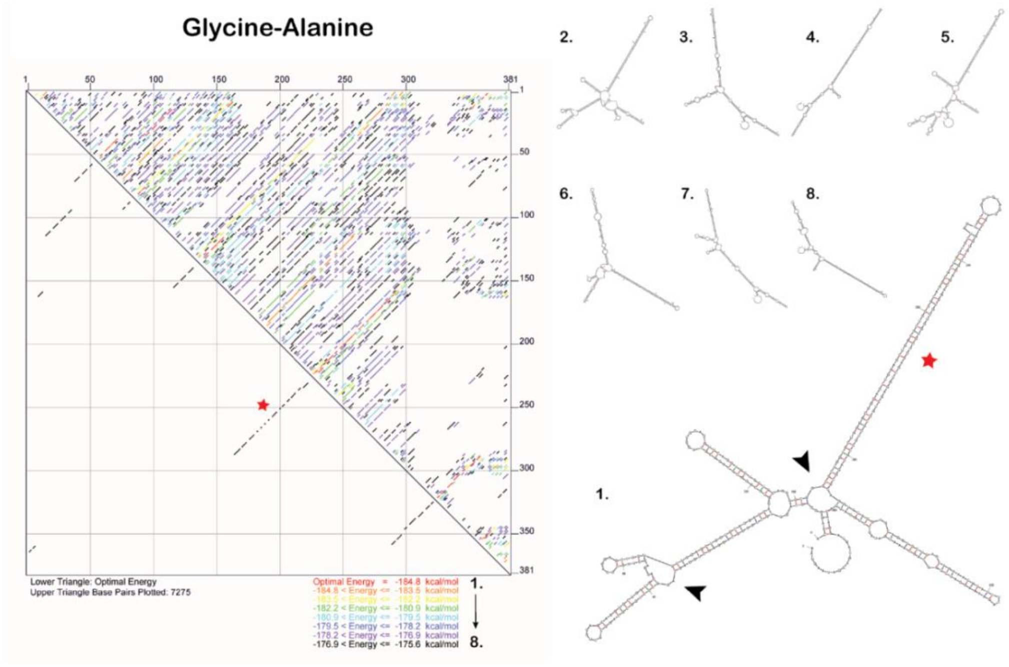

Figure S6: Generation and characterization of DPR constructs. Using manual shuffling in combination with Mfold software, codon-optimized DPR constructs were generated for each DPR species. Care was taken in minimizing G-quadruplex potential and hairpin stability. An example dot plot of GA is shown presenting the eight most likely structures arranged according to minimum free energy. The red star indicates a semi-stable hairpin in plot and structure, and arrowheads indicate destabilizing loops and bulges.

Table S1: List of all RNAi lines used for nuclear transport targeted RNAi screen.

Gene	Flybase ID	TRiP	GD	KK
Art1	CG6554	31348	40388	
Art2	CG3675		26058	109925
Art3	CG6563			109448
Art4	CG5358	31349	15645	107009
Art6	CG9927	36831	29331	107897
Art7	CG9882	36832		106639
Art8	CG16840		20305	100228
Art9	CG9929		39072	105577
Cas	CG2102		2929	100305
cdm	CG7212	31639		
CG16892	CG16892	51938	23844	101415
CG32165	CG32165		49306	109561
CG8219	CG8219		24245	103487
CG8771	CG8771		36023	102650
csul	CG3730	43200	34290	108850
Ebo	CG3923	32347	34737	
emb	CG13387	31353	3347	103767
Fbx011	CG9461	31484	24039	109599
Fs(2)Ket	CG2637		22348	107622
Kap-alpha1	CG8548		28921	108741
Kap-alpha3	CG9423		36103	106249
Karybeta3	CG1059		39713	105602
mbo	CG6819		47690	
msk	CG7935		38963	108415
Mtor	CG8274	32941	24265	110218
Ndc1	CG5857		3408	101264
Nup107	CG6743		22407	110759
Nup133	CG6958			110194
Nup153	CG4453			107750
Nup154	CG4579	34710	21878	106136
Nup160	CG4738	32391	21937	109318
Nup205	CG11943		38608	
Nup214	CG3820	33897	41964	
Nup37	CG11875		16342	109814
Nup43	CG7671		33645	108595
Nup44A	CG8722	32942	40717	106489
Nup50	CG2158	34580	20824	100564
Nup54	CG8831			103724
Nup58	CG7360			108016
Nup62	CG6251	35695	44808	100588
Nup75	CG5733		27495	
Nup93-1	CG11092	31196	16189	100315

Nup93-2	CG7262	51758	22552	
Nup98-96	CG10198	28562	31198	109279
Pen	CG4799	35439	34265	102627
ran	CG1404	31392	24835	104417
Ranbp11	CG33139	55142	44731	110496
Ranbp16	CG33180			
Ranbp21	CG12234		31706	
Ranbp9	CG5252	33004	27383	110236
RanGap	CG9999		30568	108264
ran-like	CG7815	27512	22567	109695
Rcc1	CG10480	36067	38389	110321
sec13	CG6773	32468	50367	110428
Trn	CG7398		6544	105181
Trn-SR	CG2848	25988	33569	

Grey cells indicate RNAi lines depicted in Figure 1 and Figure S3.

Table S2: Semi-quantitative assessment of hnRNPA3-positive cytoplasmic inclusions.

**hnRNPA3**	C1	C2	C9-1	C9-2	C9-3	C9-4	C9-5	C9-6
frontal cortex	-	-	+	-	+	+	+	-
dentate gyrus	-	-	+	-	+	+	+	-
cerebellum	-	-	-	-	+	-	+	-

Score: -, negative; +, <5 NCI/mm^2.

Table S3: Semi-quantitative assessment of ASYM24-positive cytoplasmic inclusions.

**ASYM24**	C1	C2	C9-1	C9-2	C9-3	C9-4	C9-5	C9-6
frontal cortex	+	-	-	+	-	+	+++	-
dentate gyrus	-	-	+++	++	++	+	++++	-
parahippocampal gyrus	-	-	+	+	+	+	+	-
cerebellum	-	-	+++	+	-	++	+	-

Score: -, negative; +, <5 NCI; ++, 5-10 NCI; +++, 11-20 NCI; ++++, >20 NCI/mm2.

Table S5: Sequences of codon-optimized constructs

GA50	GA25
ATGGGAGCAGGTGCTGGTGCAGGAGCTGGAGCAGGCGCTGGCG<br>CAGGCGCTGGAGCAGGTGCTGGTGCAGGAGCTGGCGCAGGAGC<br>TGGTGCTGGAGCTGGTGCAGGAGCAGGCGCTGGCGCAGGCGCA<br>GGTGCAGGTGCAGGTGCTGGTGCAGGTGCTGGCGCTGGTGCTG<br>GTGCTGGAGCTGGAGCTGGCGCTGGGGCAGGCGCTGGTGCTGG	ATGGGAGCAGGTGCTGGTGCAGGAGCTGGAGCAGGC<br>GCTGGCGCAGGCGCTGGAGCAGGTGCTGGTGCAGGA<br>GCTGGCGCAGGAGCTGGTGCTGGAGCTGGTGCAGGA<br>GCAGGCGCTGGCGCAGGCGCAGGTGCAGGTGCAGGT<br>GCTGGTGCAGAGCAGAAACTCATCTCAGAAGAGGAT

TGCTGGTGCAGGAGCAGGTGCCGGAGCAGGTGCTGGTGCTGGA<br>GCTGGAGCTGGCGCTGGCGCAGGCGCAGGTGCAGGTGCAGGTG<br>CTGAGCAGAAACTCATCTCAGAAGAGGATCTGGCAGCAAATGA<br>TATCCTGGATTACAAGGATGACGACGATAAGTAA	CTGGCAGCAAATGATATCCTGGATTACAAGGATGAC<br>GACGATAAGTAA
GR50	GR25
ATGGGACGTGGTCGTGGACGTGGTCGTGGACGTGGTCGAGGCC<br>GAGGTCGTGGTCGTGGTCGTGGACGAGGACGTGGACGTGGTCG<br>TGGACGAGGTCGTGGACGTGGCCGAGGACGAGGTCGTGGACGA<br>GGACGTGGACGTGGTCGTGGACGTGGTCGAGGCCGAGGTCGTG<br>GTCGTGGTCGTGGACGAGGCCGTGGTCGTGGACGTGGTCGTGG<br>ACGAGGCCGTGGTCGTGGTCGTGGACGAGGTCGTGGACGTGGC<br>CGAGGACGAGGTCGTGGACGAGGACGTGGACGAGGACGTGGA<br>CGTGAGCAGAAACTCATCTCAGAAGAGGATCTGGCAGCAAATG<br>ATATCCTGGATTACAAGGATGACGACGATAAGTAA	ATGGGACGTGGTCGTGGACGTGGTCGTGGACGTGGT<br>CGAGGCCGAGGTCGTGGTCGTGGTCGTGGACGAGGA<br>CGTGGACGTGGTCGTGGACGAGGTCGTGGACGTGGC<br>CGAGGACGAGGTCGTGGACGAGGACGTGGACGTGGT<br>CGTGGACGTGAGCAGAAACTCATCTCAGAAGAGGAT<br>CTGGCAGCAAATGATATCCTGGATTACAAGGATGAC<br>GACGATAAGTAA
PA50	PA25
ATGGCACCTGCTCCAGCACCTGCACCTGCACCTGCACCTGCTCC<br>AGCACCAGCTCCTGCTCCCGCTCCTGCACCAGCACCAGCTCCAG<br>CTCCTGCTCCTGCTCCTGCTCCTGCACCTGCTCCAGCACCAGCTC<br>CAGCACCAGCTCCTGCTCCTGCACCAGCACCTGCACCTGCACCT<br>GCTCCAGCACCTGCTCCTGCTCCTGCACCAGCACCAGCTCCTGC<br>ACCTGCTCCTGCTCCTGCTCCTGCACCAGCACCTGCACCTGCTC<br>CTGCTCCTGCTCCTGCTCCTGCTCCTGCACCTGCTCCAGAGCAG<br>AAACTCATCTCAGAAGAGGATCTGGCAGCAAATGATATCCTGG<br>ATTACAAGGATGACGACGATAAGTAA	ATGGCACCTGCTCCAGCACCTGCACCTGCACCTGCAC<br>CTGCTCCAGCACCAGCTCCTGCTCCCGCTCCTGCACC<br>AGCACCAGCTCCAGCTCCTGCTCCTGCTCCTGCTCCT<br>GCACCTGCTCCAGCACCAGCTCCAGCACCAGCTCCTG<br>CTCCTGAGCAGAAACTCATCTCAGAAGAGGATCTGG<br>CAGCAAATGATATCCTGGATTACAAGGATGACGACG<br>ATAAGTAA
PR50	PR25
ATGCCTCGTCCACGGCCACGGCCACGCCCTCGTCCACGGCCACG<br>GCCACGCCCTCGTCCTCGGCCTCGTCCACGGCCACGGCCACGCC<br>CTCGTCCTCGGCCTCGTCCTCGACCACGACCTCGACCGCGTCCT<br>CGACCTCGTCCTCGACCACGACCTCGACCGCGTCCTCGACCACG<br>TCCTCGTCCTCGGCCTCGTCCACGGCCACGGCCACGCCCTCGTC<br>CTCGGCCTCGTCCTCGACCACGACCTCGACCGCGTCCTCGACCT<br>CGTCCTCGACCACGACCTCGACCGCGTCCTCGACCACGTGAGCA<br>GAAACTCATCTCAGAAGAGGATCTGGCAGCAAATGATATCCTG<br>GATTACAAGGATGACGACGATAAGTAA	ATGCCTCGTCCACGGCCACGGCCACGCCCTCGTCCAC<br>GGCCACGGCCACGCCCTCGTCCTCGGCCTCGTCCACG<br>GCCACGGCCACGCCCTCGTCCTCGGCCTCGTCCTCGA<br>CCACGACCTCGACCGCGTCCTCGACCTCGTCCTCGAC<br>CACGAGAGCAGAAACTCATCTCAGAAGAGGATCTGG<br>CAGCAAATGATATCCTGGATTACAAGGATGACGACG<br>ATAAGTAA

Table S6: Clinical data of patient material used in this study

ID	Patient	Clinical diagnosis	Neuropathological diagnosis	Age of death
C1	Control 1	Carcinoma of pancreas.	Mild tau pathology, PART (primary age related tauopathy)	90
C2	Control 2	Acute paraplegia	Myelomalacia	81
C9-1	C9orf72 patient 1	FTD	FTLD- TDP type B	61

			P62-proteinopathy	
C9-2	C9orf72 patient 2	ALS	MND<br>TDP proteinopathy<br>P62 proteinopathy	55
C9-3	C9orf72 patient 3	FTD-ALS	FTLD-MND<br>TDP proteinopathy<br>P62 proteinopathy	51
C9-4	C9orf72 patient 4	FTD	FTLD- TDP type B<br>P62-proteinopathy	63
C9-5	C9orf72 patient 5	FTD	FTLD- TDP type B<br>P62-proteinopathy	66
C9-6	C9orf72 patient 6	ALS	P62 proteinopathy<br>Mild TDP proteinopathy	63
